# What do men and women envy each other for?

**DOI:** 10.3389/fpsyg.2024.1455199

**Published:** 2024-09-24

**Authors:** Tereza Kimplova, Michaela Krakovska, Radim Badosek, Panajotis Cakirpaloglu

**Affiliations:** Department of Educational and School Psychology, University of Ostrava, Ostrava, Czechia

**Keywords:** envy, sex difference, inequality, ablative envy, social comparison, Czech Republic

## Abstract

**Introduction:**

This study explores the phenomenon of envy directed towards the opposite sex. Envy is a complex emotion that can manifest in various forms, often reflecting societal and biological influences. Previous research suggests a potential imbalance in the domains where men and women experience envy.

**Methods:**

The research was conducted in the Czech Republic using a demographically representative sample of 1,769 individuals (867 men and 902 women). Respondents were asked what they envied about the opposite sex, and qualitative analysis was performed on their answers. A total of 32 distinct categories of envy were identified.

**Results:**

The analysis revealed significant differences between men and women in the nature of their envy. Women expressed envy towards men in domains linked to biological conditions and social advantages, such as salary, prestige, physical strength, and the absence of menstruation, childbirth, and menopause. Men, on the other hand, envied women for their physical attractiveness, ease of seduction, ability to perform traditionally female tasks, and motherhood. Some categories, such as safety and tolerance, were shared by both genders, but the underlying reasons differed. For instance, women envied men for not having to fear being alone, while men envied women for being protected by men.

**Discussion:**

These findings confirm existing evidence of a gender imbalance in envy. The study also uncovered a novel form of envy, termed “ablative envy”, which refers to the desire for the absence of an unpleasant quality. This suggests that envy between the sexes is deeply rooted in both biological and social factors, offering new insights into gender dynamics.

## Introduction

Despite the fact that contemporary society is more open-minded (Charlesworth and Banaji, 2022), sex inequality remains a significant and pressing issue (UNDP, 2018). The roles that society ascribes to us, whether indirectly or explicitly, have a significant impact on various aspects of our lives. We are informed of the roles we are expected to fulfil, the manner in which we are to behave, the way in which we are to dress, and even the emotions we are to experience (Brody, 1993). The company enforces compliance with these standards. The violation of these norms is met with social sanctions that elicit negative emotions such as shame, anger, and sadness. The qualities that are highly valued by society in one sex may be perceived as indications of weakness or inadequacy in the other (Wen et al., 2020). The reason that led us to observe these issues were narrative stories with manifestations of emotions and imbalances that we started to observe in our intervention-therapy and pedagogical practice over several years. We decided to focus more on this phenomenon, which probands named as envy. However, after studying the research and literature already conducted, we came to the conclusion that researchers were not directly addressing this topic. Based on our findings, we hypothesized that there are differences in the object and frequency of envy between men and women. The research question was straightforward: “What do you envy in the other sex?”

## Theoretical background

### Psychology of envy

Envy, a fundamental human emotion, has long been the subject of interest to both scientists and philosophers. Despite extensive study, no single definition captures all its variations. Envy’s perception and objects have evolved due to socio-economic, cultural, and religious shifts. Aristotle (in Xiang et al., 2017) provided one of the earliest systematic definitions, describing envy as an irrational and unpleasant emotion marked by feelings of inferiority, resentment, and anxiety. He identified envy as a strong dislike of another’s happiness, particularly if that person is similar or equal to us. Envy typically targets fame, honor, reputation, and desirable fortunes—especially those we wish to possess ourselves. The study of envy was initially stimulated by psychoanalysis. The most prominent proponent of this theory was its founder, Sigmund Freud, who argued that envy develops in the oral stage of an individual’s psychosexual development when oral needs are inadequately met. Another significant concept is that of penis envy, or Penisneid, which is associated with the phallic period. The concept was developed by Klein, who reworked the original theory, replacing the penis with the breast. According to Klein (2005), envy is an innate emotion and the first manifestation of the death drive. According to Hiles (2007), breast envy is a primary form of envy that, if adequately processed, does not impede further development.

Recently, there has been a growing interest in the topic of envy outside of the conventional psychoanalytic interpretation (Smith and Kim, 2007). Parrott and Smith (1993), in Xiang et al., 2017;Truong et al., 2022; Caputo, 2014; Smith and Kim, 2007; Milic, 2019 define envy as a feeling of lack of another person’s qualities, achievements, or possessions that one desires or wishes the other did not have. Kardong (1996), cited in Okholm, 2008) defines envy as a desire to possess the qualities, achievements or possessions of another individual. This differentiates it from jealousy, which is more akin to a fear of losing something one already possesses. In everyday life, jealousy and envy are often conflated, yet most scholars distinguish between the two emotions (e.g., Buss, 2013; Freud, 2000). Envy is focused on acquiring something that is not currently available or on reducing the value of something that is already possessed. In contrast, jealousy is directed towards the protection of what has already been acquired, although there is a risk of losing it (Buss, 2013). Reber and Reber (2001) posit that envy can be classified as a specific form of anxiety. In accordance with McDougall (1871–1938), envy is defined as a state of mind characterized by hostile thoughts about wealthier individuals. It is typically distinguished from envy by the involvement of a third person (Smith et al., 1999).

Buss (2013) posits that envy is a multifaceted emotion that is triggered when an individual experiences a discrepancy between their current state and the possession of something they desire. According to Crusius et al. (2021), envy as a complex emotion is also related to greed. Ramachandran and Jalal (2017) propose that envy is an emotional phenomenon that is not contingent on logic. This corroborates the theory of Aristotle’s (n.d.) that individuals with similar characteristics are more prone to envy than those with dissimilar attributes. This was demonstrated in a thought experiment (Ramachandran and Jalal, 2017), in which study participants were asked to indicate whether they envied a neighbor who was 50% richer than they were or Bill Gates more. Ten out of the 11 participants indicated that they envied the neighbor more, despite the fact that, from a logical perspective, they should have envied the richer person. This illustrates the evolutionary nature of envy, whereby individuals compete within the context of their own social status. Envy is inextricably linked to social comparison, which influences feelings of competition and the pursuit of prestige and power (Belk, 2011). An individual’s ability to elicit envy may ultimately determine their social status and significantly influence consumer behaviour.

Envy is a lifelong issue that is meant to measure oneself against a competitive environment. It is therefore unsurprising that greater envy and hatred are felt when a situation is perceived as unfair (Milic, 2019). In the work of Griffiths (2004, as cited in Ellman, 2012), it is posited that certain emotions may not manifest outwardly in an overt manner, as they are perceived to be advantageous in terms of concealment. Consequently, the distressing emotion of envy is concealed in order to prevent its outward expression, which might otherwise result in the loss of competitive advantage. In addition to this, Okholm (2008) proposes an amendment to the definition, suggesting that the envier also wishes for the envied person to lose what they envy. In his work, Okholm draws upon Schoeck (1969) to illustrate envy as a destructive force, rather than a mere theft. Van de Ven (2016) even defines envy in two variants, considering one as benign and the other as malicious. The distinction between these concepts’ hinges on the emotional responses they elicit. In contrast, benign envy is associated with a tendency to improve oneself, whereas malicious envy is linked to a motivation to bring about the downfall of the other. Crusius and Lange (2021) found that the main difference between malicious and benign envy lies not only in the emotional response, but also in the different cognitive focus, the so-called counterfactual thoughts. Those who experienced malignant envy thought more about others, whereas those who motivated benign envy manifested by thinking about themselves. In the case of envy, van de Ven et al. (2012) emphasize social determination and comparison—the more unfair a situation is perceived to be, the more individuals experience malicious envy. Lange et al. (2018a) also found that both variants of envy are related to the dark triad. Benign envy is associated with machiavellian behavior, while malicious envy is associated with both machiavellian and psychopathic behavior. Although these are character tendencies, the effects of malignant envy according to (Dong et al., 2020) can effectively limit mindfulness. Both forms of envy can be measured using the Benign and Malicious Envy Scale developed by Lange and Crusius (2015). According to the authors, each type of envy has distinct motivational origins and behavioral manifestations. Furthermore, the questionnaire has been validated in different national versions, including the Vietnamese version (e.g., Truong et al., 2022). Wobker and Kenning (2013) observed that gender influences strategic decision-making in individuals exhibiting destructive envy behavior.

In a different approach, Xiang et al. (2017) defined envy using neural correlates. The researchers employed voxel-based morphometry (VBM) imaging to identify envy. From a neurological perspective, envy is strongly manifested in areas associated with self-esteem, social perception and related emotions (Xiang et al., 2016). In a study by Lisovenko et al. (2022), it was observed that excessive activation of mirror neurons was present in individuals with a long-term and frequent experience of envy. Another neurological view of envy as a painful experience recorded by fMRI is presented by Takahashi et al. (2009). Other measurable outcomes of experienced envy are described by Izquierdo and Johnson (2007). According to the authors, envy induced by competition for resources can result in psychosomatic illnesses. As evidenced by studies conducted by Jiang and Wang (2020), envy can be used as a predictor of depression.

## Envy among sex

Freud was a prime protagonist in the study of gender differences in envy. According to Freud, women lack the capacity for independent desire and therefore must rely on the desire of a man. The manifestation of desire in women is evidenced by envy. As a result, women enter into a relationship with a man to vicariously obtain something they do not have (Benjamin, 2016), which brings us to the classic theme of penis envy. However, Benjamin presents an alternative explanation for girls’ perceived interest in the penis, based on Karen Horney (1967) view that in toddlerhood, interest is not genital in nature, but rather an organ of control, particularly in relation to urination. This purely practical explanation is also evident in our research.

In previous research, the attention of researchers has been focused on the envy of persons towards members of the same sex. Reenkola (2021) states that female envy is more hidden due to social pressure. According to the author, the key finding Reenkola (2021) argues that the topic of women’s envy towards other women is not much discussed in the literature because of the shame and guilt that may be caused by these feelings.

In recent decades, several studies have attempted to identify the objects of envy in men and women. Milic (2019) posits that envy should show gender differentiation, which is related to gender adaptation to different stimuli. This was previously confirmed by Hiles and Buss (2006). The women in the study envied women in the area of physical attractiveness, men envied men more in the area of sexual experience and more attractive partner.

The specific gender differentiation of envy was also addressed by Delpriore et al. (2012). The research indicated that men were more envious of their peers‘access to finance, possession of a status object, and academic and athletic achievement. Women exhibited greater envy of physical attractiveness, popularity, social well-being, prominent family, and superior clothing. It can be concluded that envy differs significantly between men and women, as evidenced by our pre-research. Differences in the level of perceived envy were also identified in research by de Zoysa et al. (2021), according to which men show higher levels of envy and tend to use more active/behavioral manifestations of this response, while women show more passive and predominantly emotional manifestations. Of course, we have to take into account the cultural context in this case.

The findings of the Henniger and Harris (2015) study on envy in adulthood indicated that envy is predominantly expressed between individuals of the same sex and similar age. The research indicates that envy undergoes changes over the course of life. The results indicated that as individuals mature, they become less envious of physical appearance, academic achievement, romantic relationships, and social status. Conversely, the study revealed that as people age, they become more envious of financial resources. In certain domains, such as happiness or a more favorable life in general, envy became more stable with age. The proportion of women who expressed envy was 79.4%, compared to 74.1% of men. The study concludes that the modern world facilitates the success of men, yet the researchers found no statistically significant evidence to support the hypothesis that women envy men’s privileges. Nevertheless, the data indicated that one in four women experienced envy regarding professional success, while one in five men reported envy related to physical appearance. These findings align with those of our study. In a related study, Buunk et al. (2012) presented respondents with hypothetical scenarios designed to elicit emotional reactions. Both genders were found to be envious and jealous most often because of social power and dominance.

Envy in the other sex was also detected in the research of a Suwada and Karwacki (2024) who focused on gender inequality research. Based on a qualitative analysis of women’s statements, they found that women envied men for their opportunities for self-empowerment and leisure. As a result of their precarious position in the public sphere and absolute responsibility in unpaid domestic work, women are in a poor psychophysical state. This research provides results that also support our findings and highlights the negative consequences of gender stereotypes that exacerbate gender imbalance (Love et al., 2024).

Based on the literature and research presented, we can conclude that none of the research has focused on the objects of gender envy. The aim of our study was to identify these objects of envy in a representative sample and thus verify our hypothesis. Assuming that our preliminary research identifies differences in envy between men and women, it is proposed to initiate a comprehensive research on this issue that would also conceptualize the quality of life domain.

## Materials and methods

The data for the pre-research were obtained by outsourcing a representative sociological research project to the professional agency INRES-SONES Inc. The research examined the opinions and attitudes of Czech citizens towards health issues, including healthy living, taking care of one’s own health, prevention, and so forth. The agency carries out sociological and social-psychological research and has conducted a national survey every year since 1995. Our sub-survey was designed to address the mental health domain of experiencing envy through the open-ended research question: “What do you envy in the other sex?”

To obtain the data, we used a structured interview method, i.e., a personal interview with the respondent. The data were collected by the agency’s professional interviewers. The structured question was set as an open-ended question with an unlimited number of answers and was worded as follows. The answers, which ranged from one word to several sentences, were then analysed. A total of 2,032 people were contacted, selected by random, quota sampling on a population aged 15 years and older. Due to the strict quota selection based on the stratification of the population, it does not make sense to report the average age of the population and therefore we do not report it.

However, we can confirm that the youngest respondent was 15 years old, while the oldest was 92 years old for men and 94 years old for women. Of the 2,032 individuals invited to participate in the survey, 263 (12.9%) declined to be interviewed. This resulted in responses from 1769 individuals, 867 males (49%) and 902 females (51%). This distribution is consistent with the composition of the population in the Czech Republic aged 15+. The statistical data were transcribed into electronic form by the agency, processed by SASD 1.4.15 software and submitted in anonymized form to the authors. As the data was provided by an external supplier, it was also the responsibility of that supplier to ensure that the respondents in the research had given their consent.

The empirical data collected through the interview question took the form of text. Due to the nature of the data, thematic analysis was employed as the analytical method. In the initial phase of the analysis, the researchers familiarized themselves with the individual statements, which were typically comprised of one-word utterances and phrases, with sentences being less frequent. Subsequent to the collection of the data, a process of repeated reading was undertaken, which was followed by the generation of initial codes with the objective of reducing the data to a more manageable size. The sample comprised 1,769 individuals, with a total of 2,073 responses coded. Some respondents indicated more than one object of envy, resulting in their assignment to multiple categories. The 27 instances of “I do not know” were excluded from the final calculation. The subsequent stage involved the identification of potential themes through the examination of the codes. Some themes were found to be similar and were subsequently assigned to more general themes following repeated consultations. The specific codes were reached through inductive coding, which is a bottom-up approach. The codes were analyzed and grouped into super categories (society, work, physiology), and the coded themes were organized into tables. Subsequently, the themes were subjected to further review and refinement, after which they were defined and named. The identification of the principal theme and content of the idea, as well as the creation of an appropriate nomenclature, are of paramount importance. The subsequent step is the production of a research report, which presents the analysis and results in detail. To enhance comprehension, illustrative quotations were also associated with each theme.

During the preparation of the study, the intention was to process the collected data primarily at the qualitative level. This entailed the analysis of respondents’ verbal statements and the application of descriptive statistics.

## Results

The results of the analysis are presented in Table 1. The table is divided into two sections. The first section comprises the statements “Nothing,” “Do not know,” and “Everything.” In this section, the largest group is that which selected the option “Nothing,” with a total of 864 responses (376 from women and 488 from men). The analysis of the statements, which were subsequently clustered, yielded the basic categories, which were then divided into supercategories. These constitute the second part of the table. The largest supercategory is “Society,” followed by “Physicality” and the last supercategory is “Psyche.” We reached this division by conducting a thematic analysis based on discourse analysis, which was supported by corpus analysis.

**Table 1:** Results by categorization.

	Frequency of occurrence		
Aggregated categories	Females	Males	Total
Nothing	376	488	864
Do not know	9	18	27
Everything	3	1	4
Total Society	451	245	696
Total Physicality	217	89	306
Total Psyche	57	119	176
Total	1,113	960	2,073

Table 2 presents the super category “Society.” The categories are arranged according to their frequency of occurrence. In this section, we will analyze the statements that respondents most frequently reported regarding envy towards the other sex. The category “Do not give a rip” is unquestionably the most prominent, with a clear majority of women. The typical statements made by women towards men in this category are as follows: less responsibility; peace of mind; the ability to relax and unwind; a relaxed life without worrying about problems; no responsibilities; less worry about everything; the ability to fall asleep straight away and nothing weighs on them; a comfortable life without worrying about housework and children; freedom, more time for themselves; and a lack of interest in Christmas presents. These figures and statements refer to a sex imbalance that may arise from the fact that women are expected to fulfil multiple roles, which places them at a disadvantage and affects, among other things, their leisure time and well-being. In some cases, while women expressed envy in a particular category, they also acknowledged the value of this trait in men. For example, one woman commented, “I think that kind of freedom, to do whatever you want, men are just bohemians.” She went on to describe men as “easygoing,” “taking everything lightly,” and “not making a big deal out of anything.” In the categories in which women are more prevalent, the next most frequently cited area is that of finance. Typical statements include higher remuneration, higher salaries, and more favorable financial rewards at work. It is perhaps unnecessary to comment on this overt reminder of the persisting issue of pay inequality. The category “Position in the world of work” draws its participants from a related field. Typical statements in this category include the possibility of career advancement uninterrupted by pregnancy, a better position in society and the workplace, and career benefits. The concept of envy encompasses not only social status and roles, but also physical appearance. Women often feel that society does not permit them to age naturally, which leads to envy of men who are able to tolerate many of the associated changes. These include the ability to age into beauty, the lack of the need to spend an hour in front of a mirror before leaving the house, the ability to spend time and money at the hairdresser’s, the ease of dressing requirements, the lack of judgement on their appearance, the absence of cellulite, and the ability to go without makeup to look good. This is why they are called “non-binding beauty standards.” The final category in which women express envy towards men is that of prestige. The category of “prestige” typically encompasses a number of factors, including a higher social status, greater respect, and a tendency for superiors to treat them with more respect. Additionally, women are often perceived as the stronger and more successful sex.

**Table 2:** Society results.

	Frequency of occurrence		
Code name of envied things	Females	Males	Total
Do not give a rip	189	20	209
Simpler, easier life	45	21	66
Finance (salary)	60	4	64
Beauty	4	53	57
Seduction, manipulation	3	49	52
Position in the world of work	44	4	48
Security of sex	16	23	39
None obligation standards of beauty	38	1	39
Tolerance	16	21	37
Prestige	24	8	32
“Women’s work” and housework (as pros)	0	24	24
Job specifics (lighter work…)	3	9	12
Retirement	9	0	9
Maternity leave	0	5	5
School	0	3	3
Total Society	451	245	696

Conversely, men are most envious of women’s physical beauty. The aforementioned characteristics are defined by the following statements: beauty, elegance, soft curves, the perception of women as sexy, attractiveness, rounded shapes, loveliness, and the possibility of becoming more beautiful. The category “Seduction, Manipulation” is defined by the following statements: the capacity to control and seduce others; the ability to utilise charm to one’s advantage; the concept of charm and sex appeal; the notion that men are driven by sexual desire; the capacity to influence and control others; the notion that women possess certain weapons. In contrast to the female-dominated category, the category “Women’s work and housework” for men represents a different perspective. While women perceive themselves to be disadvantaged in their position, men view this category through a different lens and envy women’s domestic skills. These include the ability to cook, the capacity to care for the household and children, the ability to maintain a clean home, and the ability to function effectively in any situation. My wife, for instance, is adept at cooking, cleaning, washing, and handling various tasks in a playful manner.

In certain categories, both sexes are represented to a similar extent. The highest-scoring category was “Simpler, Easier Life,” with higher scores for women who envy men. Men are generally perceived to have a less complicated and difficult life, as they do not have to manage the same challenges as women. Additionally, a simpler life is often seen as one where there is always someone to take care of men. Conversely, men also express envy towards women in the same category. It can be argued that women have an easier life. A woman’s ability to smile and gain almost anything is a testament to this assertion. The expectations placed upon women are not as high as those placed upon men. Another relatively balanced category is that of “Sex security.” Women have observed that they are not subject to the same level of threat as men in public spaces, such as parks, and that they do not have to worry about being attacked in the dark. Nevertheless, men are envious of women, particularly in relation to the protection and courtship they receive from men. This is evidenced by the fact that women are less likely to be physically assaulted in public spaces. The final category, “Tolerance,” was analysed with similar preferences of men and women from the supercategory “Society.” For instance, men cite the observation that women are permitted to express emotions at any time, whereas men are expected to earn and provide for their families. Women envy the ability to have a young mistress and the lack of social disapproval. They also benefit from a long-standing tradition of societal acceptance, although this has not changed.

Table 3 presents the supercategory of “Physicality.” The category with the greatest representation is “Menstruation, childbirth, and climacterium.” This category is comprised exclusively of responses from women, and is notably distinct from the other categories in that it is perceived as a desirable attribute that others lack. Furthermore, the responses are remarkably uniform. Another noteworthy category is “Motherhood,” in which men employed endearing terms, such as “bringing new life into the world,” “a woman as a giver of life,” and “the ability to raise a child in greater love.” Another category that touches on the body is that of sexuality. Although this category is heterogeneous, the responses of men are predominantly focused on the breasts and glutes. In order of importance, women mentioned the penis, citing its practicality and association with freedom. This is partly related to women’s envy in the category “Urinating standing up.” It is easier for women to urinate in nature, they can urinate where and how they want, and they can urinate standing up. However, it is also clear that there is another aspect to this phenomenon. It often appears in the context of „men being able to do “something.

**Table 3:** Physicality results.

	Frequency of occurrence		
Code name of envied things	Females	Males	Total
Menstruation, childbirth, climacterium	118	0	118
Sexual characteristics	12	40	52
Physical strength	32	1	33
To urinate standing up	31	1	32
Motherhood	0	26	26
Disease	12	3	15
Sexuality	1	10	11
Dexterity	9	1	10
Life expectancy	2	7	9
Total Physicality	217	89	306

The final major category, comprising almost exclusively statements made by women, is “Physical strength.” This category encompasses statements such as greater strength, bigger and stronger muscles, the ability to perform physically demanding tasks, and the ability to open a jar without difficulty.

Table 4 presents the “Psyche” supercategory. The category with the highest score is “Psychological uniqueness.” This category is highly selective and includes statements such as “hardworking,” “decisive,” “patient,” “talkative,” “persistent,” and “honest.” In this supercategory, two opposing categories can be identified: men envy women’s emotionality, while women envy men’s rationality.

**Table 4:** Psyche results.

	Frequency of occurrence		
Code name of envied things	Females	Males	Total
Psychological uniqueness	19	32	51
Doing multiple things at once	4	35	39
Rationality	31	6	37
Emotions, empathy, tenderness…	0	29	29
Resistance	3	17	20
Total Psyche	57	119	176

In the final category, “Doing multiple things at once,” men are the dominant gender, with the statements “Can do multiple things at once” and “Keep up with multiple things” being the most prevalent. Among the supercategories, “Psyche” is the sole one in which men express greater envy.

In order to ascertain whether women are more envious of men or men are more envious of women, it is possible to statistically calculate the absolute number of envies for women and men (frequency of objects of envy) in comparison to those who do not envy anything. The number of men who indicated that they did not experience envy was 488, while the number of women was 376. A goodness-of-fit χ2 test was conducted using a contingency table to examine the responses. The number of objects envied for men (454) and for women (728) were compared with the responses of men (488) and women (376) who reported that they envied nothing. The resulting value of χ2 was 65.619 (*p* < 0.001). This indicates a statistically significant difference in the number of objects of envy reported, with women demonstrating a greater tendency to report more objects of envy than men. Nevertheless, the effect size, as expressed by Cramer’s V (0.1791), is too small (df = 1 (0.10 < 0.30)) to permit a confident conclusion that this is a significant result.

## Discussion

The objective of our preliminary research was to map the phenomenon of envy between men and women. The qualitative data analysis yielded three supercategories: “Society,” “Physicality,” and “Psyche.” The results indicate that sex envy is present in all of these categories.

The objective of our preliminary research was to ascertain the distinctions between men and women in terms of envy. A specific question was posed to ascertain the particular aspects of the opposite sex that respondents found themselves envious of. The differing response patterns observed between men and women, both in terms of frequency and type of response, can be attributed to the efforts made to test the domain of women’s envy of men and men’s envy of women. Henniger and Harris (2015) found that envy is primarily manifested between people of the same sex and similar age, which is consistent with the claim of Aristotle’s (n.d.). However, their finding that there is no statistical evidence of women envying men’s privileges is in stark contrast to our findings. This discrepancy may be attributed to the fact that the researchers inquired about general envy, prompting respondents to provide responses pertaining to general envy.

In the context of salary envy, it is evident that women experience this phenomenon to a greater extent than men (60:4). This is also reflected in their perception of their position at work, with women reporting a greater sense of envy than men (44:4). This would suggest that women perceive themselves to be undervalued in terms of salary, and therefore desire higher salaries that are typically attributed to men. Conversely, when examining women’s attitudes and values, for instance in the medical field, where there is a notable proportional increase in the number of women, salary, career advancement, status, and prestige are more likely to be emphasised by men as reasons for pursuing this demanding profession. In contrast, women tend to prioritise social factors, such as patient care, working conditions, and relationships with superiors (Vevoda and Dobesova Cakirpaloglu, 2017). If we were to simplify the matter, it can be observed that men and women profess different values at least in this area. However, despite the different professed values, women are known to envy what men have. It is possible that this is a very specific area, and that the results from a cross-section of the population may not correspond to this phenomenon.

Financial resources also became an object of envy in the research of Delpriore et al. (2012), who, however, unlike our pre-research, focused on the difference in the object of same-sex envy. Men were more likely than women to report sending their peers, for example, access to financial resources. Based on these findings, we can state that there are categories of objects of envy that are universal.

In our research, the claims made by women in the salary envy category were, on average, 4.09 years younger than the overall sample. Nevertheless, the observed difference was not statistically significant. Nevertheless, this may indicate that salary conditions are more pronounced at a younger age. Charoensukmongkol (2018) also found support for this hypothesis, indicating that teenagers who engage with social media are more likely to experience envy. This can also manifest itself as competitiveness within the peer group.

A noteworthy finding emerged in the domain pertaining to the tendency of men to disclaim concerns and assume a disproportionate degree of responsibility, often relegating numerous responsibilities to women (e.g., childcare and housework). Concurrently, men envy women for this perceived “innate disposition.” Conversely, men and women exhibit disparate attitudes in response to specific circumstances. Conversely, women express envy towards men for not having such high standards of appearance and for being able to afford to age naturally. The question thus arises as to whether this is the result of biological differences, upbringing, or the influence of the social environment. Alternatively, it could be argued that this is a social strategy, given that there is a widely accepted thesis that beautiful people have it easier in life (Feingold, 1992). Furthermore, Benjamin (2016) asserts that men envy women’s fertility and ability to breastfeed. However, in our research, although there was also envy among men in the area of motherhood, it is not a mass phenomenon. Nevertheless, it can be confirmed that a certain proportion of men do indeed admire this ability and are able to envy it.

Concurrently, it is postulated that envy of the absence of biological female processes may be indicative of a problem of self-acceptance of the biologically inevitable. It is not possible to ascertain the reasons for this phenomenon with certainty. In our preliminary research (Kimplova and Lackova, 2020), we found that women envy the absence of these physiological processes in men, yet they do not wish to change them. It is evident that these processes are an inherent aspect of their lives. However, they aspire for men to also experience these processes, which is analogous to what is referred to as “malicious envy” (van de Ven, 2016) or “schadenfreude” (Takahashi et al., 2009). This possibility cannot be automatically dismissed and merits further consideration as a potential avenue for future research. It could provide further insight into the reasons and experiences associated with this atypical area.

It is similarly paradoxical that men envy women’s breasts. It must be reiterated that this does not imply that all men who envy women’s breasts aspire to possess them. It is more probable that they desire to possess them under their control and accessibility. Alternatively, it could be a form of imprinting, whereby memories of the mother are associated with the satisfaction of early physiological needs, feelings of security, and so forth. It would be beneficial to investigate this further through additional research.

## Limits and future research

The limitations of our study lie not in its representativeness, which is a common problem, but in the fact that it is a nationally homogeneous sample, which may have different characteristics from foreign samples. A further limitation is the method of data collection, which is based on a single question and therefore unable to aggregate individual responses on a similar topic and create clusters of factors.

## Conclusion

The findings of a representative survey indicate that globally, women are statistically significantly more envious than men. However, in terms of effect size, this result is not particularly robust. The social sphere is the domain in which women experience the greatest degree of envy. This is largely driven by a perception that men have an easier life, with fewer concerns and higher salaries and status at work. Conversely, men express envy towards women in this area due to their perceived beauty, seductive abilities, and manipulative skills. In the domain of physiology, women again exhibit greater levels of envy than men. This is pertinent to areas such as physical strength and the capacity to urinate in a standing position. The area of psychological qualities exhibits the lowest level of envy, with men displaying significantly greater levels of envy than women. This is particularly evident in regard to qualities such as emotional stability and the ability to perform multiple tasks simultaneously.

Our research has yielded an interesting finding in the field of physiology that challenges the conventional understanding of envy as defined by classical sources, including the work of Aristotle’s (n.d.) and Parrott and Smith (1993). In our research, we have detected a new kind of envy, which we have named ablative envy. Ablative envy, in contrast to pre-existing categories of envy, consists of envying something that one does not physiologically possess and wants to avoid. Women envy men for the absence of a condition that, in women’s experience, makes life experientially unpleasant. This is a purely emotional issue, not a conative or cognitive issue. In this particular case, women usually envy men the absence of menstruation, childbirth and menopause. An extended definition by Parrott and Smith would be as follows: Envy can be defined as a feeling of lack of another person’s qualities, accomplishments, or possessions that one desires or wishes the other person did not have. Additionally, he may wish that he did not have to endure something that the other person is spared. It is our contention that this represents a significant advancement in our understanding of the phenomenon of envy and contributes to the existing body of research on the characteristics of this emotion. This research has led to the emergence of two distinct categories of envy: “malignant envy” and “benign envy” (Belk, 2011; Lange and Crusius, 2015; van de Ven et al., 2009; van de Ven, 2016; Lange et al., 2018b).

Empirical research has demonstrated that envy of the opposite sex is not a uniform phenomenon. The fact that such differentiated envy occurs is indicative of the existence of something to envy about the other sex and that equality has not yet been achieved. The existence of sex-differentiated envy as an internal experience may therefore be taken to indicate that it is a reliable indicator of the general state of society. Our research has identified the areas in which it is felt. The specific statements and categories identified can be a stimulus for applied measures, for example in therapeutic or pedagogical practice and especially in the social and political sphere. Because some of the abundantly represented categories (envy towards salary, security, tolerance, etc.) can be reduced by social change.

In the repeated presentation of who envies whom, it is important to note that more than 42% of women do not envy men at all, and for men it is more than 56%. Although this may be the least interesting aspect of the research from a scientific perspective, it is encouraging from a psychological standpoint that, despite the continued existence of numerous gender-based differences, there are also numerous areas of common ground, which can be viewed in a positive light.

## Data Availability

The raw data supporting the conclusions of this article will be made available by the authors, without undue reservation.
